# Influence of Fermented-Moutai Distillers' Grain on Growth Performance, Meat Quality, and Blood Metabolites of Finishing Cattle

**DOI:** 10.3389/fvets.2022.874453

**Published:** 2022-05-09

**Authors:** Qiming Cheng, Duhan Xu, Yulian Chen, Mingming Zhu, Xueyin Fan, Maoya Li, Xiaolong Tang, Chaosheng Liao, Ping Li, Chao Chen

**Affiliations:** ^1^College of Animal Science, Guizhou University, Guiyang, China; ^2^Key Laboratory of Animal Genetics, Breeding and Reproduction in the Plateau Mountainous Region, Ministry of Education, Guizhou University, Guiyang, China

**Keywords:** fermented-Moutai distillers' grain, finishing cattle, growth performance, meat quality, amino acid, blood metabolites

## Abstract

The present study evaluated the effect of dietary supplementation with fermented-Moutai distillers' grain (FMDG) on the growth performance, meat quality, amino acid composition and blood metabolites of finishing cattle. Thirty cattle (2 years old; 237.55 ± 10.72 kg) were randomly assigned to one of three dietary supplementations: 0% FMDG (control), 15% FMDG (R1) and 30% FMDG (R2) [dry matter (DM) basis]. After 60 days, the inclusion of FMDG had no significant (*p* > 0.05) effects on the growth performance indices (dry matter intake, average daily gain and feed efficiency), meat quality (cooking yield, shear force, L^*^, a^*^, and b^*^ values) or bovine blood biochemical indicators (except albumin and immunoglobulin A). Cattle fed R1 had the lowest (*p* = 0.001) loin eye area. Supplementation with FMDG significantly (*p* < 0.05) increased the beef contents of various amino acids (except isoleucine and arginine) compared with the control diet. Specifically, R2 significantly increased (*p* < 0.05) the total amino acid, essential amino acid, non-essential amino acid and umami amino acid contents in beef, while the difference in bitter amino acid content between different treatments was not significant (*p* = 0.165). These results suggest that it is feasible to include FMDG at up to 30% DM without affecting the growth performance, meat quality or blood metabolites of finishing cattle.

## Introduction

Currently, feedstuff deficiency is the greatest constraint on livestock development in China (1). The appropriate use of byproduct feed resources is a viable solution to the feed crisis. Distillers' grain (DG), a cereal byproduct of ethanol production, has high nutritional value because of the removal of starch during the fermentation process and the consequent increase in digestible fiber, protein, and fat; China generates ~100 million tons of DG each year (2, 3). DG is used as a source of protein and energy due to its high protein and fat contents (4). Reis et al. (5) found that feeding dried DG improved the growth performance and meat quality of bulls. However, Crane et al. (6) reported that adding DG to the diet of lambs had no effect on growth performance and meat quality but improved the level of volatile fatty acids in the rumen. Thus, the effect of DG on the performance of ruminants is not consistent.

DG's high moisture content causes it to easily deteriorate and makes it inconvenient to transport and store (7–9). DG is usually dried; however, artificial drying significantly raises the price of DG, and excessive heating during drying also renders it more susceptible to protein damage and reduced amino acid availability (9–11). Therefore, the majority of DG is discarded or fed fresh with a limited time period (12). Previous studies found that microbial fermentation considerably improves the nutritional value of DG and extends its shelf life (13, 14). Therefore, microbial fermentation may be an alternative way of preserving DG.

Kweichow Moutai is one of the world's most famous liquors, with an annual yield of ~110,000 tons of DG. The dry matter, crude protein, neutral detergent fiber, and acid detergent fiber contents of Moutai DG are 30.46%, 21.84% DM, 36.85% DM, and 24.72% DM, respectively, according to our preliminary analysis, and the amino acid content is high, indicating that Moutai DG is a good feed resource. Previous studies found that fermented distillers' grains contain probiotic culture, prebiotic and postbiotic properties that are useful for growth performance (15). Numerous studies have examined the nutritional value of dry DG and its effects on livestock feeding and found that adding an appropriate amount of DG to the diets of cattle did not affect feed efficiency, growth performance and meat quality (16–19). Because DG is less expensive than whole grains, feeding finishing diets supplemented with 10–80% DM has become a common practice (20). Higher amounts of DG (>40% DM) in the diet, however, diminish the energy value and dry matter intake of the feed (21, 22). Yet, little information on the use of fermented Moutai DG (FMDG) as a feed source for cattle is available. The objective of this research was to evaluate the effects of finishing diets containing intermediate rates of FMDG (15 and 30% DM) on the growth performance, meat quality, amino acid content and blood biochemical index of Guanling finishing cattle. We hypothesize that adding FMDG to replace part of the concentrate in cattle diets will improve feed efficiency and meat quality.

## Materials and Methods

### Sample Preparation and Animal Ethics

Thirty Guanling cattle from Guizhou Cattle Industry Group Co., Ltd. were used in this study. The DG used in this study was obtained from the Kweichow Moutai Group in Moutai Town, Renhuai City of Guizhou Province, China. The main ingredients of Moutai DG are distilled sorghum and wheat that are a byproduct of the brewing processes. Moutai DG was fermented with biological starter for 15 days in a silo to generate FMDG. The ingredients of the biological starter were lactic acid bacteria, yeast, *Bacillus, Bifidobacterium, Clostridium butyricum*, amylase, protease, cellulase, and lipase.

All trial procedures were implemented according to the regulations of Guizhou University's Animal Care Advisory Committee (EAE-GZU-2020-T03T).

### Animals, Diets, and Experimental Design

This study was conducted at the Yueyawan cattle farm in Guanling County, Anshun City, Guizhou Province (105°58′E, 25°98′N). The test animals used in this study were Guanling cattle, which is a local breed of Guizhou Province. In 2016, the Ministry of Agriculture of China approved the implementation of geographic indication registration and protection of agricultural products (No. AGI2016-03-1987). Thirty 2-year-old Guanling steers (237.55 ± 10.72 kg) were chosen from the experimental herd and kept in three different pens (60 m^2^) with 10 stalls in each pen; each stall was provided with a feeder and a drinking trough. The three pens (containing 10 steers each) were randomly assigned to one of three dietary treatments: 0% FMDG (control), 15% FMDG (R1, replace 15% of the concentrate), or 30% FMDG (R2, replace 30% of the concentrate) (DM basis, dietary supplementation). Feed was provided as a total mixed diet on an *ad libitum* basis with a forage-to-concentrate ratio of 60:40. The dietary composition and nutrient levels are shown in Table 1. The study began on September 20, 2020 and lasted 70 days. Beef cattle were fed the experimental diets during a 10-day acclimation phase before being gradually introduced to the diets for 60 days of growth performance.

**Table 1:** Composition and nutritional content of experimental diets varying in levels of fermented-Moutai distillers' grain (FMDG).

**Items**	**Control**	**R1**	**R2**	**FMDG**
**Ingredient, % DM inclusion**
*Pennisetum Sinese* Roxb	60	60	60	
Groundcorn	20	10	0	
Soybean meal	6	3	0	
Rapeseed meal	4	2	0	
Wheat bran	8	8	8	
FMDG	0	15	30	
Supplement				
Rock fine	1.4	1.4	1.4	
Salt	0.05	0.05	0.05	
Calcium bisulfate	0.42	0.42	0.42	
Sodium sulfate	0.08	0.08	0.08	
Microelement additive	0.05	0.05	0.05	
Total	100	100	100	
**Chemical analysis**
Dry matter (%)	70.58	70.68	71.20	31.50
Gross energy (MJ/kg DM)	15.76	16.60	16.70	18.69
Crude protein (% DM)	11.40	12.64	13.88	21.88
Neutral detergent fiber (% DM)	54.47	55.47	56.47	36.56
Acid detergent fiber (% DM)	29.37	32.96	36.54	23.33
Ether extract (% DM)	3.30	3.67	4.03	6.52
Total P (% DM)	0.72	0.74	0.77	0.76
Total K (% DM)	1.22	1.15	0.95	0.45
Calcium (% DM)	0.82	0.83	0.83	0.43
**Amino acid (% DM)**
Aspartic acid	0.45	0.56	0.75	2.87
Threonine	0.22	0.30	0.40	1.39
Serine	0.25	0.31	0.46	1.89
Glutamic acid	0.85	1.26	2.13	4.23
Proline	0.24	0.38	0.63	3.49
Glycine	0.26	0.34	0.47	1.44
Alanine	0.35	0.55	0.87	2.26
Valine	0.26	0.36	0.52	1.62
Methionine	0.10	0.13	0.19	0.85
Isoleucine	0.21	0.30	0.42	1.43
Leucine	0.42	0.63	0.96	3.94
Tyrosine	0.23	0.29	0.36	1.53
Phenylalanine	0.34	0.43	0.56	1.93
Lysine	0.21	0.26	0.31	1.02
Histidine	0.35	0.42	0.50	0.92
Arginine	0.18	0.22	0.31	1.55

*FMDG, fermented-Moutai distillers' grain; Control, 0% FMDG; R1, 15% FMDG; R2, 30% FMDG; DM, dry matte*.

### Data and Sample Collection

Every morning before feeding, the refusals were weighed. Dry matter intake (DMI) (kg/d) was computed daily as the difference between feed offered and the amount of refusals (DM basis). Feed and refusal materials were sampled weekly for further detection of nutritional indicators. All samples were dried in an oven at 65°C for 48 h before being powdered through a 0.20 mm screen (FW100; Taisite Instrument Co., Ltd., Tianjin, People's Republic of China). All experimental cattle were fasted for 16 h and weighed at the beginning and end of the experimental period to determine the initial body weight (IBW) (kg) and final body weight (FBW) (kg), and average daily gain (ADG) (kg/d) was calculated using the following formula: ADG (kg/d) = (FBW – IBW)/experimental period (60 days). The feed efficiency (G:F) of each animal was calculated based on the mean ADG (kg/d) and mean DMI (kg/d). Before cattle were weighed on day 60, blood samples were taken from the jugular vein (15 mL each) and centrifuged (10 min, 1,500 × g) to prepare the serum, which was immediately stored at −20°C for blood biochemical index analysis. At the end of the trial, all experimental cattle were transferred to the slaughterhouse, where they were sacrificed *via* electrical stunning followed by jugular vein exsanguination. For additional research, samples of the longissimus lumborum muscle were taken from the cadavers on the right side of the vertebrae and frozen at −20°C.

### Laboratory Analysis

Dry matter (DM) content was determined by oven drying at 65°C to constant weight. The crude protein, ether extract, phosphorus (P), potassium (K), gross energy, and amino acid contents of the diets were determined using the AOAC (23) method. The neutral detergent fiber and acid detergent fiber contents were analyzed with a modified procedure using an ANKOM 2000 Fiber Analyzer (ANKOM Technology Corp., Fairport, NY, USA) (24).

At the slaughterhouse, the loin eye area was immediately drawn on a transparent vinyl plate and the area was measured using a planimeter (Super Planix α Planimeter, Tamaya Technics Inc., Tokyo). Cooking yield, shear force, pH, and meat color [lightness (L^*^), redness (a^*^) and yellowness (b^*^)] of the longissimus lumborum muscle were determined according to the method of Zhao et al. (25). Briefly, cooking yield was calculated using the weight of meat before and after cooking. Shear force was measured using a Warner–Bratzler shear instrument (G-Lerec.MFG. Co., USA). The pH value was measured 24 h after slaughter by a digital pH meter (STARTED 100/B, OHAUS, Shanghai, China). The color of the longissimus lumborum muscle was evaluated using a colorimeter (WSC-S, Shanghai, China). The amino acid composition and proportions in the longissimus lumborum muscle were determined by reversed-phase high-performance liquid chromatography (HPLC) using the Pico Tag method according to the method described by Rubio (26). In brief, the longissimus lumborum muscle sample was hydrolysed in 6 M HCl at 110°C for 24 h. The hydrolysate was then diluted, dried and derivatized, and the sample (10 μl) was injected onto a 300 × 3.9 mm NovaPak C18 (Waters) HPLC column.

Serum beta hydroxybutyrate (β-HB) was analyzed with an electronic β-HB meter (Precision Xceed, Abbott Diabetes Care Ltd.) (27). The concentrations of serum total protein (TP), albumin (ALB), urea nitrogen (UN), alanine aminotransferase (ALT), aspartate aminotransferase (AST), and alkaline phosphatase (ALP) were determined by using commercial kits (Beijing Sinouk Institute of Biological Technology, China). The amounts of serum immunoglobulin A (IgA), IgG, and IgM were determined by using ELISA kits (Nanjing Jiancheng Bioengineering Institute, Nanjing, China).

### Statistical Analysis

Data on the growth performance, meat quality, amino acid content and blood biochemical indices were analyzed using a one-way analysis of variance (ANOVA) to evaluate the effects of FMDG. The differences between means were assessed using Duncan's multiple range method. The effect was considered significant when *p* < 0.05. The analyses were conducted using IBM SPSS Statistics 25.0 (SPSS, Inc., Chicago, IL). The results are presented as the mean ± SEM.

## Results and Discussion

### Growth Performance and Meat Quality of Cattle

A few studies have examined the effects of FMDG in beef cattle diets on their growth performance. The growth performance of cattle is shown in Table 2. Compared with the control, dietary supplementation with R1 and R2 had no significant (*p* = 0.671) effect on DMI. Previous studies evaluating the effect of DG fed to cattle reported no effect on DMI when 200 g/kg dried-sorghum DG replaced corn (16). However, negative effects on DMI were observed when dried-sorghum DG replaced up to 450 g/kg (DM basis) sorghum grains in growing diets (22). Therefore, the addition of FMDG had no negative effect on DMI in our study because the added amount was relatively moderate (0–30%). The initial body weight of the three groups was similar, ranging from 235.33 to 241.00 kg. Adding R1 and R2 had no significant effect (*p* > 0.05) on the ADG, FBW, and G:F ratio of cattle compared to the control group, indicating that FMDG had no negative influence on growth performance. A similar observation was reported by Beretta et al. (17), who found that adding dried-sorghum DG (0–450 g/kg) to cattle diets had no effect on the ADG, FBW, or G:F ratio compared to control diets (0% DG, DM basis). Simeone et al. (18) discovered that increasing the amount of dried-sorghum DG up to 300 g/kg (DM basis) in sorghum grain-based diets had no effect on the G:F ratio of cattle. The ADG (0.65–0.75 kg/d) and G:F (0.06–0.07) values in our study, on the other hand, were lower than those (ADG, 1.44–1.62 kg/d; G:F, 0.132–0.148) reported by Beretta et al. (17). This could be because our study was conducted during the cold season (September to December), when low temperatures limit the rate and efficiency of beef cattle production (28).

**Table 2:** Effects of different dietary levels of fermented-Moutai distillers' grain (FMDG) on the growth performance of Guanling cattle.

**Items**	**Control**	**R1**	**R2**	***p*-value**
Dry matter intake (kg/d)	11.40 ± 0.27	11.14 ± 0.11	11.34 ± 0.20	0.671
Initial bodyweight (kg)	241.00 ± 20.5	236.33 ± 6.89	235.33 ± 4.76	0.946
Final bodyweight (kg)	282.50 ± 21.65	281.17 ± 8.66	274.17 ± 3.47	0.899
Average daily gain (kg/d)	0.69 ± 0.02	0.75 ± 0.04	0.65 ± 0.07	0.436
Feed efficiency	0.06 ± 0.00	0.07 ± 0.00	0.06 ± 0.00	0.321

*Control, 0% FMDG; R1, 15% FMDG; R2, 30% FMDG; DM, dry matte*.

The meat quality of cattle is shown in Table 3. The loin eye area is a useful measure of muscle growth and carcass meat content since it is closely related to carcass weight (29). In our study, feeding R1 resulted in a smaller (*p* = 0.001) loin eye area compared to that of the control and R2 groups (Table 3). The value of the loin eye area initially decreased and then increased as the FMDG addition level increased; the cause of this change must be investigated further. In this study, adding FMDG (R1 and R2) had no effect on the cooking yield, shear force, or pH of any tested muscle (*p* > 0.05) when compared to the control diet. Meat color is another important parameter of meat quality and is particularly affected by dietary factors (30, 31). pH is the main factor affecting meat color and is negatively correlated with lightness, although the mechanism underlying this relationship remains unknown (32, 33). According to Khliji et al. (34), values for L^*^ below 34 and a^*^ below 9.5 are considered dark and unsatisfactory to ordinary customers. In this investigation, no significant (*p* > 0.60) group differences were discovered in L^*^, a^*^, or b^*^ within the range of consumer acceptability, which is consistent with the findings of Nade et al. (19), who showed that feeding Holstein steers 15% dry DG had no effect on flesh color.

**Table 3:** Effects of different dietary levels of fermented-Moutai distillers' grain (FMDG) on the meat quality of Guanling cattle.

**Items**	**Control**	**R1**	**R2**	***p*-value**
Loin eye area (cm^2^)	68.5 ± 0.29^a^	53.00 ± 3.06^b^	70.67 ± 1.45^a^	0.001
Cooking yield (%)	60.18 ± 0.34	65.62 ± 1.04	63.55 ± 3.27	0.229
Shear force (*N*)	65.37 ± 6.57	56.15 ± 4.90	65.37 ± 4.12	0.387
pH	6.80 ± 0.09	6.96 ± 0.06	6.80 ± 0.18	0.581
**Color**
L*	34.87 ± 0.01	35.99 ± 1.08	34.56 ± 0.66	0.601
a*	9.84 ± 0.27	10.31 ± 1.06	9.93 ± 0.64	0.706
b*	17.84 ± 0.11	17.80 ± 0.11	17.84 ± 0.18	0.971

*Means within the same row (a, b) with difference superscripts differ significantly from each other (p < 0.05).*

Control, 0% FMDG; R1, 15% FMDG; R2, 30% FMDG; L*, lightness; a*, redness; b*, yellowness.

### Amino Acid Profiles of Cattle Meat

Meat is a key source of vital dietary amino acids and protein. Furthermore, certain amino acids improve the flavor and palatability of meat (35). Amino acids are the most basic building blocks of proteins, and their type and composition are among the most essential indicators of protein concentration and distribution, which directly affect the nutritional value of beef (36). In this study, a total of 16 amino acids were detected in all treatment groups, among which aspartic acid (Asp), glutamic acid (Glu), leucine (Leu), and lysine (Lys) were the dominant amino acids in beef (Table 4), indicating that the meat of Guanling cattle was rich in amino acids. Few studies have examined the effect of adding DG to the diet on the amino acid content of beef. Our study showed that the addition of FMDG significantly (*p* < 0.05) increased the content of various amino acids [except isoleucine (Ile) and arginine (Arg)] in beef. This was because the contents of various amino acids were significantly greater in diets supplemented with R1 and R2 than those in control diets (Table 1). The Ile and Arg contents in beef from the R1 and R2 groups were higher than those in the control group, but the difference was not significant, indicating that different amino acids in feed have varied conversion efficiencies (37). Another possible reason is that FMDG contain probiotics that bind with epithelial cells of stomach and bowels and help to synthesize the amino acids which result in improving the content of amino acids in beef (15, 38, 39). We detected 17.13–21.23 g/100 g of total amino acid (TAA) content in beef; including 7 essential amino acids (EAAs): threonine (Thr), valine (Val), methionine (Met), Ile, Leu, phenylalanine (Phe) and Lys at 7.09–8.68 g/100 g, and 7 non-essential amino acids (NEAAs): Asp, serine (Ser), Glu, proline (Pro), glycine (Gly), alanine (Ala), and tyrosine (Tyr) at 8.24–10.22 g/100 g (Table 4). The addition of R2 significantly (*p* < 0.05) increased the contents of TAA, EAAs, and NEAAs in beef compared with the control group. According to the FAO/WHO/UNU (40), the EAA/TAA ratio in good-quality meat should be ~40%, and the EAA/NEAA ratio should not be <0.60. The EAA/TAA ratio in beef in our study was 40.38–41.39%, while the EAA/NEAA ratio was 0.84–0.86, indicating that the beef was of good quality. The composition and concentration of umami amino acids (UAAs) and bitter amino acids (BAAs) in beef were investigated to determine the impact of FMDG on beef flavor (41). In our study, 4 kinds of UAAs (Asp, Glu, Gly, and Ala) and 4 kinds of BAAs (Val, Met, Ile, and Leu) were detected; the UAA content of beef supplemented with R2 was significantly higher than that of the control (*p* = 0.046), while the difference in BAA content between different treatments was not significant (*p* = 0.165). Overall, adding FMDG to the diet altered the proportion and composition of amino acids in beef, thus improving its flavor, with 30% FMDG having the best effect (42).

**Table 4:** Effects of different dietary levels of fermented-Moutai distillers' grain (FMDG) on the amino acid content of beef.

**Amino acids (g/100 g beef)**	**Control**	**R1**	**R2**	***p*-value**
Aspartic acid^∧#^	1.65 ± 0.12^b^	1.95 ± 0.09^a^	2.06 ± 0.01^a^	0.032
Threonine*	0.83 ± 0.05^b^	0.97 ± 0.04^a^	1.03 ± 0.01^∧^	0.027
Serine^∧^	0.67 ± 0.05^b^	0.80 ± 0.03^a^	0.85 ± 0.01^a^	0.030
Glutamic acid^∧#^	2.80 ± 0.29^b^	3.38 ± 0.20^a^	3.50 ± 0.05^a^	0.012
Proline^∧^	0.69 ± 0.04^b^	0.88 ± 0.03^a^	0.87 ± 0.02^a^	0.009
Glycine^∧#^	0.75 ± 0.05^b^	0.87 ± 0.04^ab^	0.90 ± 0.01^a^	0.054
Alanine^∧#^	1.03 ± 0.08^b^	1.13 ± 0.05^ab^	1.24 ± 0.03^a^	0.094
Valine*&	0.92 ± 0.06^b^	1.05 ± 0.04^ab^	1.09 ± 0.01^a^	0.071
Methionine*&	0.52 ± 0.03^b^	0.60 ± 0.03^ab^	0.66 ± 0.00^a^	0.015
Isoleucine*&	0.89 ± 0.07	1.01 ± 0.05	1.05 ± 0.02	0.122
Leucine*&	1.55 ± 0.10^b^	1.77 ± 0.07^ab^	1.88 ± 0.02^a^	0.046
Tyrosine^∧^	0.65 ± 0.04^b^	0.78 ± 0.04^a^	0.81 ± 0.00^a^	0.027
Phenylalanine*	0.78 ± 0.04^b^	0.93 ± 0.03^a^	0.96 ± 0.01^a^	0.016
Lysine*	1.61 ± 0.10^b^	1.89 ± 0.08^a^	2.00 ± 0.03^a^	0.028
Histidine	0.65 ± 0.08^b^	0.85 ± 0.02^a^	0.91 ± 0.01^a^	0.025
Arginine	1.15 ± 0.07	1.36 ± 0.07	1.27 ± 0.13	0.356
Total amino acid	17.13 ± 1.26^b^	20.33 ± 0.68^a^	21.23 ± 0.09^a^	0.035
Essential amino acids (EAAs)	7.09 ± 0.46^b^	8.21 ± 0.35^ab^	8.68 ± 0.09^a^	0.038
Non-essential amino acids (NEAAs)	8.24 ± 0.65^b^	9.78 ± 0.48^ab^	10.22 ± 0.04^a^	0.045
EAA/NEAA	0.86 ± 0.01	0.84 ± 0.01	0.85 ± 0.01	0.435
Umami amino acids	6.23 ± 0.53^b^	7.33 ± 0.38^ab^	7.69 ± 0.02^a^	0.046
Bitter amino acids	3.87 ± 0.30	4.43 ± 0.19	4.68 ± 0.28	0.165

*Means within the same row (a, b) with difference superscripts differ significantly from each other (p < 0.05).*

*Control, 0% FMDG; R1, 15% FMDG; R2, 30% FMDG.*

^*^Essential amino acids (7 essential).

*^∧^Nonessential amino acids (7 essential).*

*^#^Umami amino acids (4 essential).*

*^&^Bitter amino acids (4 essential)*.

### Blood Biochemical Indices of Cattle

The blood biochemical indices of cattle are shown in Table 5. Our research showed that adding FMDG had no effect (*p* > 0.05) on bovine blood biochemical indicators [except albumin (ALB) and immunoglobulin A (IgA)]. The levels of ALB increased at first and then decreased as the FMDG concentration increased: the level of ALB in the R1 group was significantly (*p* = 0.049) higher than that in the R2 group, whereas the level of IgA in the control group was significantly (*p* = 0.044) higher than that in the R1 group.

**Table 5:** Effects of different dietary levels of fermented-Moutai distillers' grain (FMDG) on the blood biochemical index of Guanling cattle.

**Items**	**Control**	**R1**	**R2**	***p*-value**
Beta hydroxybutyrate (mmol/L)	3.87 ± 0.07	3.74 ± 0.17	3.59 ± 0.22	0.112
Total protein (g/L)	58.08 ± 1.72	65.14 ± 3.92	63.76 ± 3.78	0.343
Albumin (g/L)	23.84 ± 0.22^ab^	24.06 ± 0.12^a^	23.36 ± 0.18^b^	0.047
Urea nitrogen (mmol/L)	2.11 ± 0.12	2.20 ± 0.04	2.13 ± 0.07	0.172
Alanine aminotransferase (U/L)	22.4 ± 0.65	21.71 ± 1.20	22.57 ± 1.88	0.143
Aspartate aminotransferase (U/L)	64.33 ± 4.08	64.71 ± 0.84	62.96 ± 2.04	0.158
Alkaline phosphatase (U/L)	125.87 ± 2.43	123.45 ± 4.83	124.91 ± 4.59	0.156
Immunoglobulin A (g/L)	4.79 ± 0.18^a^	4.29 ± 0.06^b^	4.53 ± 0.06^ab^	0.048
Immunoglobulin G (g/L)	8.42 ± 0.05	9.15 ± 0.49	9.17 ± 0.25	0.246
Immunoglobulin M (g/L)	2.16 ± 0.11	2.26 ± 0.13	1.98 ± 0.08	0.279

*Means within the same row (a, b) with difference superscripts differ significantly from each other (p < 0.05).*

*Control, 0% FMDG; R1, 15% FMDG; R2, 30% FMDG*.

The beta hydroxybutyrate (β-HB) content in blood, plasma, or serum is the most commonly used index to diagnose hyperketonaemia in cattle, with a critical value of 1.0–1.4 mmol/L (43, 44). The normal level of β-HB in bovine blood is generally <1.0 mmol/L. There was no significant (*p* = 0.112) difference in the β-HB levels of any groups in this study, and all were within the normal range (17.95–19.34 μmol/L).

Serum concentrations of total protein (TP), albumin (ALB) and urea nitrogen (UN) can accurately reflect protein metabolism and feed protein utilization efficiency. UN, on the other hand, is commonly measured clinically as a method of determining non-protein nitrogen levels and discovering various kidney diseases. All groups had similar concentrations of TP and UN with increasing FMDG concentrations (*p* > 0.05). The UN level shows a positive linear association with dietary DMI and ruminal degradability (45), which explains why the UN concentrations in our study groups were similar. This indicates that the addition of FMDG to the diets did not negatively impact the efficiency of feed protein utilization or the kidney function of cattle. However, as the concentration (15–30%) of FMDG increased, cattle ALB levels decreased. This contrasted with results from Kim et al. (46), who found that adding Korean rice wine residue (10–15% DM) to Hanwoo steer diets had no effect on the ALB levels in the blood. This indicated that supplementation with more than 15% FMDG may affect the protein metabolism and immunity of cattle.

Alanine aminotransferase (ALT), aspartate aminotransferase (AST) and alkaline phosphatase (ALP) are important indicators of liver function. There were no significant (*p* = 0.112) differences in the ALT, AST, and ALP levels of any groups in our study, which indicates that adding an appropriate amount of FMDG (<30%) to the diets did not impact the liver function of cattle. A similar result was obtained by Obeidat (47), who reported that adding dried-corn DG (0–150 g/kg DM) to Awassi lamb diets had no effect on ALT, AST and ALP levels when compared to the control diet.

Immunoglobulin A (IgA), IgG, and IgM are serum immune indices that are extremely important for three kinds of immunoglobulins in mammals. All groups had similar concentrations of IgG and IgM as the FMDG concentration increased (15–30%, *p* > 0.05), but the level of IgA first decreased and then increased (*p* = 0.044). This indicates that adding an appropriate amount of FMDG to the diets could increase the IgA level in bovine serum. A similar result was found by Weber and Kerr (48), who reported that adding corn DG (35% DM) to pig diets increased serum IgA. In terms of mechanism, the consumption of DDG can improve humoral immunity through the activity of β-glucans (48, 49).

Overall, adding an appropriate amount of FMDG to the diets of Guanling cattle had no detrimental impact on blood metabolites, kidney functions, or liver functions, showing that this byproduct is safe and does not create any health issues. Even though there were no negative effects of feeding 0–30% DM FMDG in our study, we recommend monitoring blood metabolites, kidney function, and liver function, especially when cattle are fed FMDG for extended periods of time (47).

## Conclusions

The results of this study indicate that dietary supplementation of Guanling cattle kept feedlots with up to 30% DM FMDG did not affect growth performance, meat quality or blood metabolites; however, this supplementation altered the amino acid composition and proportions in beef, improving its flavor. However, as the ALB concentration decreased, more research in this area is needed to ensure that cattle protein metabolism and immunity are not affected. During prolonged FMDG feeding, we recommend that blood metabolites, kidney, and liver function are closely monitored.

## Data Availability Statement

The original contributions presented in the study are included in the article/supplementary material, further inquiries can be directed to the corresponding author/s.

## Ethics Statement

The study was conducted according to the guidelines and approval of the Guizhou University's Animal Care Advisory Committee (EAE-GZU-2020-T03T).

## Author Contributions

PL and CC designed the experiments and revised the manuscript. QC, DX, YC, XF, ML, XT, and CL performed the experiments. QC and DX wrote the manuscript. PL and QC carried out the data analysis. All authors reviewed and considered the manuscript. All authors contributed to the article and approved the submitted version.

## Funding

This work was financially supported by funds from the National key research and development subject (2021YFD1300302), Guizhou Talent Base of Grassland Ecological Animal Husbandry (RCJD2018-13), Guizhou Province Science and Technology Plan Project ([2020]3009), Guizhou Provincial Department of Education Project ([2017]059), and Guizhou University Cultivation Project (Guida Renji Hezi [2020]9).

## Conflict of Interest

The authors declare that the research was conducted in the absence of any commercial or financial relationships that could be construed as a potential conflict of interest.

## Publisher's Note

All claims expressed in this article are solely those of the authors and do not necessarily represent those of their affiliated organizations, or those of the publisher, the editors and the reviewers. Any product that may be evaluated in this article, or claim that may be made by its manufacturer, is not guaranteed or endorsed by the publisher.
